# Methotrexate-Induced Accelerated Nodulosis: A Case Series

**DOI:** 10.31138/mjr.08424.mia

**Published:** 2024-12-31

**Authors:** Ramaswamy Subramanian, Nikita Chettri, Rahul Bisaralli, Purna Bansa, Mahabaleshwar Mamadapur

**Affiliations:** 1 Department of Clinical Immunology and Rheumatology, JSS Medical College, JSS Academy of Higher Education and Research, Mysuru, India,; 2 Department of Pharmacy Practice, JSS College of Pharmacy, JSS Academy of Higher Education and Research, Sri Shivarathreeshwara Nagar, Mysuru, Karnataka, India,; 3 Department of Clinical Immunology and Rheumatology, SDM College of Medical Science and Hospital, Dharwad, Karnataka, India,; 4 JSS Medical College, JSS Academy of Higher Education and Research, Mysuru, India

**Keywords:** human leukocyte antigen, hydroxychloroquine, sulfasalazine, rheumatoid arthritis, subcutaneous nodules

## Abstract

Methotrexate-induced nodulosis, also known as methotrexate-induced accelerated nodulosis (MIAN), is a rare side effect of methotrexate therapy. Methotrexate (MTX) is commonly used to treat various autoimmune diseases, such as rheumatoid arthritis, psoriasis, and inflammatory bowel disease. In this case series, we present patients with MIAN, discussing their clinical features, diagnostic approaches, and management strategies. We aim to increase recognition of this rare side effect of MTX therapy, facilitate early diagnosis, and improve clinical management, thus minimising the burden of this debilitating complication on affected individuals.

## INTRODUCTION

Methotrexate is an immunosuppressive agent, commonly used to manage various autoimmune diseases such as rheumatoid arthritis (RA), psoriasis, and inflammatory bowel disease.^1^ However, MTX therapy can cause a range of side effects, from mild gastrointestinal symptoms to severe hepatotoxicity and bone marrow suppression. One of the uncommon complications associated with MTX is accelerated nodulosis, characterised by the rapid development of subcutaneous nodules resembling rheumatoid nodules which is the most common extra-articular manifestation, primarily affecting the hands and feet.^2^ MIAN usually presents as painful or tender nodules that develop in the subcutaneous tissue, particularly on the extremities. These nodules are usually smaller and more numerous than rheumatoid nodules.^3^ Although the pathogenesis of MIAN is unclear, immunological dysregulation and drug-induced tissue damage have been suggested as possible causes.^4^

Management of MIAN typically involves discontinuation of methotrexate therapy, which often leads to the resolution of the nodules. In some cases, alternative therapies may be necessary for the underlying condition being treated with methotrexate.^5^ Despite its rare occurrence, awareness of this complication is crucial for timely diagnosis and appropriate management to reduce potential morbidity and optimise patient outcomes.

## CASE NARRATION

### Case 1

**Figure 1.**
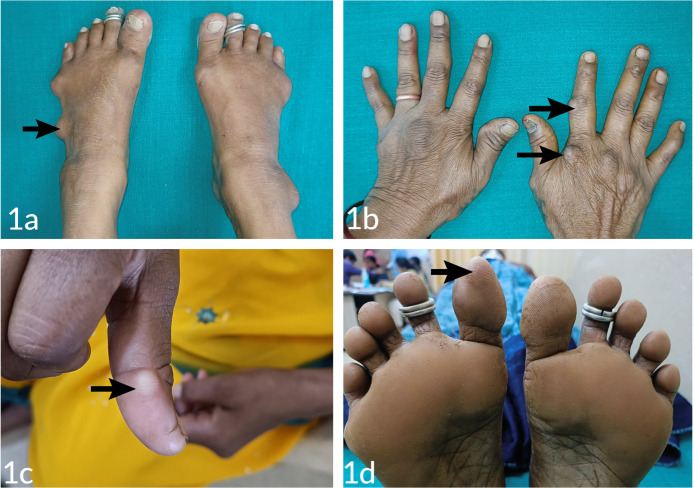
Multiple nodules (black arrows) over the base of the 5th toe **(A)**, proximal interphalangeal metacarpophalangeal joints of the hands **(B)**, thumb **(C)**, and great toe **(D)**.

A 51-year-old female with a 10-year history of seropositive RA initially was started on MTX 15mg per week. After eight years of treatment, she presented with painless, nodular swelling over multiple joints, including meta-carpophalangeal (MCP), proximal interphalangeal (PIP), distal interphalangeal (DIP), metatarsophalangeal (MTP), midfoot, and thumb. Physical examination showed deformities of both hands and feet typical for RA (**Figure 1A–B**). Multiple painless subcutaneous nodules were present over the above-mentioned sites. However, there were no swollen and tender joints. Her C-reactive protein (CRP) and erythrocyte sedimentation rate (ESR) were normal, and the Disease Activity Score (DAS) was 2.52 suggesting remission. A diagnosis of MIAN was made, and she was started on Hydroxychloroquine (HCQ) tablets with no improvement in symptoms. The clinical details are summarised in **Table 1**.

**Table 1.** Clinical characteristics and outcomes of patients with MIAN.

**Sl. No.**	**Age/Gender**	**Disease**	**Drug involved**	**Treatment Duration**	**Nodules appeared after**	**Action taken**	**Outcome**
**1**	51/F	RA	MTX	10 years	8years	MTX was withdrawn, and no treatment started	Not resolved
**2**	35/M	RA	MTX	2 years	3 months	MTX continued, SSZ started	Reduction in nodule size
**3**	56/F	RA	MTX	5 years	8 months	MTX was withdrawn, and HCQ started	No new growth
**4**	49/F	RA	MTX	8 years	6 years	MTX continued, and no treatment started	No new growth
**5**	28/F	RA	MTX	3 years	2 years	MTX continued, Leflunomide started	No new growth

### Case 2

A 35-year-old male with seropositive RA for 2 years presented with painless nodules over the thumb and PIP joint after three months of treatment with MTX 15mg per week (**Figure 1C**). The tender joint count (TJC) and swollen joint count (SJC) were 6 and 4, respectively. The MTX was continued because of moderate disease activity (DAS 28- 4.76) and was initiated on Sulfasalazine (SSZ). A reduction in the size of the nodules was noted during follow-up.

### Case 3

A 56-year-old female patient with a 5-year history of RA, positive for both Rheumatoid Factor (RF) and Anti-Citrullinated Protein Antibody (ACPA) presented with painless nodules over the base of the 1st toes after 8 months of treatment with MTX. On examination, nodules were found over the base of the 1st toe. Her inflammatory markers had increased slightly, and DAS suggested moderated disease activity (DAS28-4.7). MTX was stopped, and she was initiated on HCQ. No new growth was seen.

### Case 4

A 49-year-old female with a history of RA for 8 years, positive for both RF and ACPA had been on oral MTX 15mg per week ever since. She presented with painless nodules over the right wrist-ulnar side, 1st toe, MTP, and the base of the 5th toe after 6 years of treatment (Figure 1D). Clinical examination showed elevated ESR, one swollen, and three tender joints. MTX was continued, and no treatment was started. No new growth was seen.

### Case 5

A 28-year-old female with 3 years of seropositive RA presented with painless nodules over the thumb and 5th toe MTP after 2 years of MTX therapy. Skin biopsy confirmed rheumatoid nodules which were later changed to MIAN. DAS28 was 4.8 suggesting moderate disease activity. MTX was continued, and Leflunomide was initiated. No new growth was seen.

**Figure 1** shows multiple nodules (black arrows) over the base of the 5^th^ toe (**Figure 1A**), proximal interphalangeal metacarpophalangeal joints of the hands (**Figure 1B**), thumb (**Figure 1C**), and great toe (**Figure 1D**). Table 1 shows the Clinical characteristics and outcomes of patients with MIAN.

## DISCUSSION

MIAN is a significant concern in clinical practice, particularly for those undergoing long-term methotrexate therapy for autoimmune disorders such as RA and psoriasis. Our research supports previous findings on MIAN, shedding light on its intricate development and clinical significance.

Kremer and Lee reported accelerated rheumatoid nodulosis in 1986 while doing a long-term MTX therapy study for RA.^6^ The exact mechanisms underlying the development of MIAN are still not fully understood. Although the adenosine signaling pathway is currently the leading hypothesis to explain the effectiveness of methotrexate in RA patients,^7^ the precise cascade of events that lead to MIAN formation requires further clarification. In a study by Merrill et al., it was suggested that methotrexate induces adenosine production from infiltrating monocytes, which then stimulates the adenosine A1 receptor and enhances giant cell formation in vitro.^3^ The researchers concluded that drugs inhibiting adenosine A1 receptors might be useful for treating methotrexate-induced accelerated nodulosis.^8^

The studies show that MIAN can occur in around 8%–11.6% of patients being treated for RA. The cumulative methotrexate dosage varies significantly, ranging from 90 to 7200 mg. Instances have been reported where the time between starting the treatment and the onset of nodulosis ranged from 3 months to 12 years. Predisposing variables include male gender, smoking, high RF and ACPA titers, severe arthritis, and HLA predisposition (HLADRB1).^9^ This delayed onset highlights the importance of long-term monitoring for patients taking MTX, even if they don’t show obvious clinical symptoms. Early detection of MIAN is crucial as it allows for timely intervention to reduce the risk of potential complications, such as impaired joint function and cosmetic disfigurement.

The clinical presentation of MIAN varies widely, ranging from asymptomatic subcutaneous nodules to painful, ulcerating lesions. MIAN presents diagnostic challenges as it can resemble other subcutaneous lesions such as rheumatoid nodules or malignancies. Although clinically, MIAN is smaller than rheumatoid nodules and develops more rapidly in soft tissue and away from the joints, usually involving the fingers. MIAN tends to occur while arthritis is inactive during methotrexate treatment.^10^ Therefore, histopathological examination remains the gold standard for definitive diagnosis, enabling differentiation from other nodular skin conditions.

Agarwal et al. observed that out of fourteen cases, four patients in their study recovered spontaneously despite continuing MTX treatment, which was similar to the fourth patient in our study.^3^ However, in our case, no new growth was observed, and the reason for this is unclear and requires further study.

Ahmed et al. studied HLA association retrospectively in twenty-one patients of RA with MIAN. Those with HLA class II allele DRB1*0401 were genetically predisposed to MIAN.^9^ MIAN occurred usually 3 years after starting MTX, regressed in 6 months if MTX is promptly discontinued, and can persist for 3.5 years if MTX is continued.^10–11^

Ana G. Palmeiro et al. did a literature review of 109 cases from 38 studies. The average age of the patients was 52.7 years. Of the 53 cases with accessible gender information, 65% were female. Most patients (n = 99, 90.8%) had RA, in addition to also having dermatomyositis, scleroderma, juvenile idiopathic arthritis, psoriatic arthritis, systemic lupus erythematosus, and aortitis. The mean duration of RA was 12 years. Among 27 RA patients tested for HLA-DR04, 19 (70.4%) were positive. Out of 84 RF tests, 80% were positive, while 10 out of 11 ACPA tests were positive (91%). The majority had low disease activity. Subcutaneous nodules were seen in 96 (88.1%) individuals, primarily in the fingers (n = 66, 60.6%), olecranon process (n = 22, 20.2%), and dorsum of hands (n = 18, 16.5%). The most common culprit agent was MTX, followed by etanercept, tocilizumab, leflunomide, azathioprine, letrozole, and infliximab. TX treatment duration before developing accelerated nodulosis ranged from a few months to 12 years. HCQ, colchicine, systemic corticosteroid, D-penicillamine, azathioprine, leflunomide, etanercept, cyclophosphamide, cyclosporine, topical corticosteroids, and rituximab were administered to 47 (43.11 percent) of the patients despite the suspension or continuation of the culprit drug.^5^

The management of MIAN involves a multidisciplinary approach that integrates rheumatological, dermatological, and surgical expertise. Although stopping methotrexate is often the first step, it may not always be enough to stop the progression of MIAN. Additional treatments such as colchicine, sulfasalazine, hydroxychloroquine, or d-penicillamine have been found to reduce the size of the nodules, even if methotrexate treatment is continued.^12–14^ In some cases, surgical removal of the nodules is an option for relief of symptoms or for cases that do not respond to other treatments, but it comes with the risk of recurrence and wound complications.

## CONCLUSION

MIAN represents a clinically significant complication of MTX therapy, necessitating vigilance in patient monitoring and management. Further research into the pathophysiology and therapeutic strategies for MIAN is imperative to optimise patient outcomes and mitigate disease burden in individuals receiving MTX for autoimmune disorders.
